# Co-creating an arts-based eye health education strategy in Zanzibar: process, outcomes and lessons learnt

**DOI:** 10.1136/bmjgh-2022-009317

**Published:** 2022-09-06

**Authors:** Ving Fai Chan, Dina Belluigi, Ai Chee Yong, Damaris Mulewa, Pirindhavellie Poonsamy-Govender, Christine Graham, Eden Mashayo, Ronnie Graham, Carlos Price-Sanchez, Fatma Omar

**Affiliations:** 1 Centre for Public Health, Queen's University Belfast, Belfast, UK; 2 College of Health Sciences, University of KwaZulu-Natal, Durban, South Africa; 3 School of Social Sciences, Education and Social Work, Queen's University Belfast, Belfast, UK; 4 Critical Study of Higher Education Transformation, Nelson Mandela University, Port Elizabeth, South Africa; 5 Partnership for Child Development, Imperial College London, London, UK; 6 Tanzanian Optometry Association, Dar es Salaam, Tanzania; 7 Vision Aid Overseas, London, UK; 8 Ministry of Health, Zanzibar, Tanzania

**Keywords:** public health, health education and promotion, health services research

## Abstract

**Introduction:**

Published examples of health programme co-creation are scarce and we found none in the field of eye care. We described the outcomes and lessons learnt from the ZANZIbar Arts for Children’s Eyesight (ZANZI-ACE) eye health programme co-creation process.

**Methods:**

We used a 2.5-day stakeholder workshop (number of participants=34) to develop the ZANZI-ACE intervention, which aimed to use music performances in eye health education to improve child eye health service uptake in Zanzibar. A Zanzibar-wide music competition was then launched to encourage local participation, followed by a judging session to select three pieces as the ZANZI-ACE eye health programme intervention materials.

**Results:**

The barriers to the improved uptake of child eye health services raised by the participants were mainly cultural and social. Sensitising parents, teachers, children and community members with proper eye health knowledge was the key to addressing these barriers. The goal of sensitisation is to improve children’s vision so that they can achieve their fullest potential. Music and song ranked highest among the proposed art forms, so three music pieces were chosen as the ZANZI-ACE eye health programme intervention materials. A detailed ZANZI-ACE implementation strategy, a theory of change and key performance outcomes indicators were developed.

**Conclusion:**

The co-creation process and outcomes of the ZANZI-ACE eye health programme show that engaging a diverse group of stakeholders is critical to developing locally relevant health programmes. The lessons learnt from the process will prove useful to researchers who aspire to design innovative health programmes.

**WHAT IS ALREADY KNOWN ON THIS TOPIC**

- Even though co-creation strategies can effectively develop context-specific interventions that promote health services uptake, there is limited literature on the co-creation process and outcome and none in the eye health sector.

**WHAT THIS STUDY ADDS**

- This study reports the outcomes and lessons from an arts-based eye health education co-creation process which can guide similar health intervention development.
- Local stakeholders identified the barriers to service uptake and produced music materials and detailed implementation strategies.

**HOW THIS STUDY MIGHT AFFECT RESEARCH, PRACTICE OR POLICY**

- The process and lessons learnt from co-creating the ZANZI-ACE (ZANZIbar Arts for Children’s Eyesight) interventions could contribute to the knowledge pool to inspire researchers in other health sectors to develop innovative health programmes that are adaptive to the local context.

## Introduction

Eye health education has received less attention and funding than disease prevention and treatment initiatives.^1^ A Cochrane review^2^ and The Lancet Global Health Commission on Global Eye Health^3^ concluded that more codesign studies are required to promote sustainable behavioural changes among communities and children with eye problems. Published examples of health programme co-creation are scarce^4^ and none related specifically to eye care. However, the co-creation of health interventions is not a new concept. The intervention developed through this co-creation process is critical to addressing complex public health issues faced in eye health. By tailoring the intervention to the needs of the local communities, solutions can be more effective and sustainable.^5^

The strength of these interventions comes from the diversity of stakeholders who collaboratively design interventions and implementation strategies that are holistic, appropriate, sustainable and scalable for solving a health problem of interest.^6–8^ Such stakeholders may be planners, implementers, beneficiaries, community members and/or policymakers. Stakeholders are selected strategically based on their roles, knowledge and networks to ensure the fitness-for-purpose of the co-creation and implementation processes.

Zanzibar, a predominantly rural location with many infrastructural challenges, has about 22 000 children (5% of the island’s children aged 6–12 years old) in need of conjunctivitis treatment and spectacle correction (Ministry of Health Zanzibar monitoring data. 2018, unpublished). National policy and programmes have been devised to address this, with the Zanzibari government working to improve public health practices and access to eye health services (Eye Health Strategic Plan 2018–2022). Despite this, a case study conducted in 2017 showed that about 42% of children in rural Zanzibari communities who needed a pair of eyeglasses did not have them.^9^ Challenges to uptake may have stemmed from underlying suspicions of Western medicine and public health initiatives, as well as the more prevalent use of traditional, less effective, healing methods.^10^

Implementing eye health education campaigns before outreach eye screening programmes has successfully increased service uptake in other Sub-Saharan African settings.^1^ Chan *et al*^11^ costing analysis of a school eye health programme showed that about 46% of the screening kits were dedicated to printing information, education and communication (IEC) materials. These IEC materials, including booklets, posters and brochures to engage children in Zanzibar at schools, have been negatively received and these posters and brochures were often destroyed.^12^ Combined with poor use of eyeglasses and compliance due to teasing from peers and misconceptions on eyeglasses-wearing habits, the local stakeholders urged planners to improve the existing eye health education strategy to overcome these barriers.^12^

Health-focused, arts-based interventions have catalysed behavioural changes globally.^13–15^ A review by Bunn *et al*^15^ showed that HIV/AIDS programmes had used arts-based methods widely in Sub-Saharan Africa and most notably using theatre and musical approaches. Boyce *et al*^14^ showed that published studies on arts-based health interventions predominantly employed music (76.8%) and highlighted the value of arts in various healthcare settings. In Africa, arts-based health interventions are mainly built on traditional oral and performance methods.^16^ For instance, highly positive results of the health promotion campaigns in The Gambia, where traditional music was incorporated to improve Ebola awareness, were documented.^17^ The *kanyeleng* musical performers contributed to an inclusive model based on cultural strengths, as opposed to the traditional top-down approaches that may instil fear and suspicion. Instead, it was found that they ‘promote positive emotions of love and happiness over anger and fear’.^17 18^

The main barrier to eyeglasses uptake in mainland Tanzania^19^ and Zanzibar^20^ was that eyeglasses were not being prioritised by the local communities and that traditional medicine would be their preferred choice of treatment. Among children, the fear of being teased by peers was the main barrier.^12^ Further engagement with local communities revealed that these communities perceived traditional healers as the first point of contact for treatment and a previous arts-based, peer-delivered eyecare education intervention in rural mainland Tanzania was developed to address the barrier.^21^ This intervention significantly enhanced the community’s eye health knowledge by 6.3%, leading to a - four times increase in monthly service uptake.^21^ This non-traditional intervention attracted the attention of Zanzibar health authorities and they indicated a strong commitment to codevelop an arts-based education initiative. This led to the ZANZIbar Arts for Children’s Eyesight (ZANZI-ACE)^22^ strategy to address the local sociocultural barriers to eyecare service uptake.

Recognising that ZANZI-ACE would be a new approach for Zanzibar, the research team acknowledged that engaging key local and intergenerational stakeholders would be essential to co-creating culturally appropriate strategies. Of primary importance was that these strategies must be responsive to local barriers, ensure local acceptability and be developed with proper implementation plans. Through multisectoral partnership and co-creation workshops held in 2020, we sought to identify the local barriers, needs, attitudes and behaviours regarding the potential utilisation of an arts-based approach to increase the uptake of child eye health services in Zanzibar. In the ZANZI-ACE context, we adapted Rimer and Kreuter’s^5^ co-creation definition as the collaborative development of eye health intervention by local ministry stakeholders, professional bodies, non-governmental organisations, artists, art groups and academics.

The overall aim of the ZANZI-ACE project is to determine the effectiveness of using arts-based eye health education to improve the uptake of child eye health service in Zanzibar. However, this paper only describes phase 1 of the project, which is the process of community engagement and public authority involvement, and the project’s outcomes and the lessons learnt. These outcomes included (1) informed understandings of and proposed solutions to address sociocultural barriers to the uptake of child eye health services from the perspectives of multiple stakeholders, (2) stakeholders’ preferences for the content of eye health messages and the arts-based form for the eye health education strategy, and (3) development of the intervention and implementation strategy, underpinned by an agreed theory of change.

## Methods

### Ethical considerations

Local permission to conduct the workshops was obtained from the Ministry of Health in Zanzibar. All persons who participated in the workshop provided active (opt-in)^23^ informed consent (of adults older than 18 years old) and assent (of minors 18 years and younger). We first contacted the school principals and the parents of potential children to be invited to the workshop and explained the workshop’s aim. Once the parents provided their consent, we explained to the children the aim of the workshop and then asked them if they were willing to participate in the workshop. If they agreed to participate, we obtained their assent in the presence of their parents and teachers. Extra care was taken to ensure discussions with children were conducted in an environment where they would feel safe to express their opinions freely. This included using trained facilitators who were experienced in working with children and providing the children ample time to prepare and present their opinions. This approach was guided by the toolkit published by Save the Children: *So you want to consult with children? A toolkit of good practice*.^24^ To ensure equitable partnership among collaborators, all researchers contributed one way or another to study conceptualisation, research management, data acquisition and analysis and data interpretation, acknowledged as authors, received training, and their participation protected by the local principal investigator and research team (see reflexivity statements in online supplemental appendix 1).

10.1136/bmjgh-2022-009317.supp1 Supplementary data

### Patient and public involvement

This co-creation process is part of the ZANZI-ACE patient and public involvement strategy. As described in this article, the public and the local key stakeholders (including children who were beneficiaries of the school eye health programme) were invited to attend the co-creation workshops and judge the music competition entries. Local artists participated in a Zanzibar-wide music competition.

### Setting

Zanzibar is part of the United Republic of Tanzania, with autonomy in domestic affairs. It consists of two islands, Unguja and Pemba, which have both commonalities and differences in their cultural practices. About 97% of the population are Muslims, with a distinctive music tradition such as sung poetry (Taarab).^25^ This African-Muslim music is influential in Zanzibar, the coastal area in Tanzania and Kenya.^25^ Children under 18 years form 48% (826 848)^26^ of the Zanzibari population of about 1 717 608.^27^ Pemba has a smaller population (less than 350 000), with 80% living in rural areas.^27^

### Participants

The Steering Group first brainstormed who key stakeholders might be and from an initial list of approximately 60 stakeholders, before narrowing it down to 34 key stakeholders drawn from eye health, education, culture, civil society and the community. The group excluded those who were not representative or unwilling and unable to represent the stakeholders. To identify information-rich representatives from children, parents and teacher groups, we consulted the principals of the schools which had been enrolled in the previous school eye health programme in Zanzibar. From both Unguja and Pemba islands, 34 participants were purposively recruited.^28^ We included participants from health groups, education groups, artistic groups and community groups (table 1). Of central importance was the inclusion of local stakeholders from both islands (21 from Unguja and 13 from Pemba) to ensure diversity among those involved in the ZANZI-ACE programme design and material development and the beneficiary groups well represented.

**Table 1:** Characteristics of participants in the ZANZI-ACE workshop

Participant groups	Participants, male/female (n)	Unguja/Pemba participants (n)	Total number of participants
Health groups			
Ministry of Health, Social Welfare, Elderly, Gender and Children	2/1	2/1	3
Eye hospitals and facilities	3/0	1/2	3
Non-governmental organisations	2/1	2/1	3
Education groups			
Ministry of Education and Vocational Training	2/1	1/2	3
Teachers	2/1	1/2	3
Principals	3/0	2/1	3
Artistic groups			
Ministry of Culture, Information and Communication	0/2	2/0	2
Council of Art, Film Censorship and Culture	1/1	2/0	1
Arts and culture groups	1/1	1/1	2
Media	2/0	2/0	2
Community groups			
Community leaders	1/1	1/1	2
Parents	2/1	2/1	3
Children	1/2	2/1	3
Total	22/12	21/13	34

ZANZI-ACE, ZANZIbar Arts for Children’s Eyesight.

During the workshop interactions, participants were divided into six heterogeneous groups, which included children so that their opinions were represented. Each group consisted of four to six participants from different participant groups to encourage discussions.

### Co-creation methods and proceedings

Developing the ZANZI-ACE eye health education strategy involved (1) the co-creation workshop and follow-up discussions, (2) a Zanzibar-wide music competition and (3) a feedback meeting. We employed the workshop methods^29^ as they helped to focus on the core issues and used the purposively selected groups that could provide insights that prioritised the participants’ needs. Understanding the inherent weaknesses of the workshop methods in maintaining equal power dynamics and equal contribution, we complemented them with a careful selection of facilitators and participants (participatory action approach), designing a discussion guide and employing qualitative data analysis. We recruited two trained facilitators (FO, female; EM, male) to conduct the 2.5-day workshop (2 full days from 20 to 21 April 2021 and half day on 22 April 2022) in Zanzibar to allow for full and rich discussion. The discussion topics were guided by the domains listed in the Implementation Science Research Development (ImpRes) tool and guide^30^ (table 2). We provided participants with background documents (summaries of national health and education policies, information from child eye health studies and arts-based intervention, translated into Swahili) in advance to accelerate the process. On the first day of the workshop, we facilitated the participants’ identification of key barriers to children’s eye health and proposed solutions and the expected results if the proposed solutions were to be successfully implemented. On the second and third day, we discussed the possibilities of different local art forms and how arts-based methods could be operationalised in different ways for knowledge transfer and exchange. Following the last day of the workshop, ongoing discussions were conducted with local stakeholders to finalise the ZANZI-ACE intervention, implementation strategy and indicators used to measure its success.

**Table 2:** Topics discussed during the workshop and the judging criteria for the music pieces

Topics discussed during the workshop	Judging criteria for the music pieces
Have there been any reported cases of eye problems among children in your community/schools? If yes, what eye conditions were found among the children? What are the main reasons children do not access eye health services in your community? What art forms can you think of to effectively share child eye health messages to the community? For each art form, consider any advantages and challenges (ethical issues, literacy levels, cultural sensitivity, cost, sustainability, etc) that we might face. What key messages must be conveyed to the community about child eye health to improve service uptake? What is needed to implement Zanzibar’s arts-based eye health education strategy programme? What do you want to achieve in Zanzibar’s arts-based eye health education strategy programme? What are the concerns you have about the arts-based possibilities discussed? And, how may they be addressed?	Content appropriateness The relevance of thematic content. Appropriate for use among children and community. Culturally acceptable content. The key messages were comprehendible. Lyrical quality Included key messages as instructed in the competition brief. Lyrics that can capture the interest of children and adults. The audience can easily understand the lyrics. Artistic value Emotional melody with cadences (surprises). Melody is relatable to the audience. Rhythm well executed.

### Workshop notes analysis

A framework analysis^31^ was used to analyse the process documentation and workshop notes. The workshop notes were captured primarily in Swahili or occasionally English if the participants preferred to speak or write in English to ensure accuracy. FO and EM made additional remarks or analytical notes (eg, clarifications from the groups on short phrases provided during feedback sessions and responses from the groups when questions were raised) during the workshop to provide further context to the discussion. All meeting notes in Swahili were later translated into English by our bilingual facilitator (FO) and back-translated by our Swahili-speaking analyst (DM). To ensure trustworthiness^31^ and not be influenced by the perspectives and viewpoints of one analyst, we included three analysts from different backgrounds: ACY is a public health optometrist, DM is a school health programme specialist and VFC is a global health practitioner.

The three analysts (ACY, DM and VFC) read the meeting notes and familiarised themselves with the notes before the coding began. The analysts created a blank table for each topic from the question guide populated with relevant quotes from workshop notes (one quote per row), for example topics exploring barriers to service uptake, preferred art forms and proposed key messages. Starting with one workshop note, two analysts (ACY and DM) independently populated the tables and wrote a brief statement about each quote, adopting an inductive approach to capture unexpected responses. Thereafter, they conferred and reached a consensus on this work and then independently assigned condensed themes based on the meaning statements. After coding the first two meeting notes, ACY and DM compared and agreed on a set of codes to use for the remaining meeting notes. The process was repeated until no new codes and subcodes emerged. VFC considered both opinions in cases where disagreement persisted. Initial themes were reviewed multiples times to identify the similarities. Where possible, the initial themes were merged. For example, six initial themes were obtained when determining the barriers to service uptake. When reviewing the initial themes and after discussion with the third analyst, we condensed three initial themes (ie, lack of awareness, lack of felt need and others) into one (ie, lack of awareness or felt need for eye treatment). The final theme was then generated (ie, misconception on the need for eye treatment). Frequency distribution was used to rank the preferred art forms and key messages. Subsequently, a matrix was developed in Excel, summarising the data by different topics. The findings were then used to understand the barriers to child eye health service uptake, proposed solutions to address sociocultural barriers to service uptake, stakeholders’ preferences for the content of eye health messages and the arts-based form for the eye health education strategy (see online supplemental appendix 2 for the thematic diagram of the main key themes and subthemes). They also informed the theory of change map using the analysis framework.

10.1136/bmjgh-2022-009317.supp2 Supplementary data

### Music competition and judging process

Subsequently, a Zanzibar-wide music competition was launched from 1 May to 30 June 2021. The competition was advertised via television broadcasts, local newspapers and invitations to local art groups. Participants were informed that their musical pieces would be selected by a panel of technical and music experts and stakeholder representatives based on predefined assessment criteria. Two weeks after the closing of the competition, a feedback meeting was conducted. A judging panel was formed based on their artistic expertise, understanding of entertainment value and lyrical content, and their ability to determine the musical pieces’ appropriateness and relevance. The panel consisted of 30 representatives, who met to review the submitted musical pieces (table 3).

**Table 3:** Profiles of judges of the Zanzibar-wide music competition

Judges	Profile
Judges 1 and 2	Two award-winning local musicians with expertise in traditional Zanzibari music and contemporary music.
Judges 3 and 4	Music broadcasters from local popular radio stations.
Judges 5–8	Parents from Unguja (n=2) and Pemba (n=2) islands.
Judges 9–11	Two children from Unguja (aged 8 and 15) and one child from Pemba (aged 12).
Judge 12	One child (aged 10 years) from Unguja who is a musician.
Judges 13–16	Two ophthalmic clinical officers and two education officers.
Judges 17–20	Two primary school teachers and two secondary school teachers.
Judges 21–22	Two respected traditional healers in Zanzibar.
Judges 23–30	Two village leaders, two religious leaders and four community members.

A briefing session was conducted with the panel before the judging session to ensure the judges understood the process. Objective judging is done by individual members scoring each musical piece based on content appropriateness, lyrical quality and artistic value on a 10-point scale (1 being not good and 10 being excellent). They ranked the pieces based on the final score (out of 900). They also gave justifications for their ratings to ascertain their subjective artistic judgements from a diverse panel group. The three pieces with the highest ranking were selected as winners to be commissioned for use as part of the eye health education strategy materials.

## Results

### Barriers to uptake of child eye health services, proposed solutions and expected outcomes

Four themes were identified as barriers to uptake of child eye health services in Zanzibar. These barriers were (1) religious belief, (2) social stigma on spectacle wear among children, (3) misconception on the need for eye treatment and (4) the belief in traditional medicines (table 4).

**Table 4:** Barriers raised by the participants and accompanying excerpts from the workshop notes

Themes	Illustrative excerpts
Religious beliefs	“Other people regard that having certain eye conditions emerged due to curses or punishment from God.”
	“Some say eye problems result from witchcraft - being bewitched by someone!”
	“If you get an eye problem - it is God’s wish!”
Social stigma on the use of eyeglasses among children	“There is peer influence against wearing eyeglasses and fear of distorting their facial appearance.”
	“They are shy about putting on their eyeglasses due to negative thoughts of being seen as disabled or someone special.”
	“Children get teased when they wear their eyeglasses.”
Misconception on the need for eye treatment	“Some people believe that wearing eyeglasses will increase more eye problems in children, so they ask their children not to wear them.”
	“Children at a young age are prohibited from wearing eyeglasses, thinking that their eyes will be spoiled earlier.”
	“You are born with it (eye problems) naturally.”
	“No need to see the doctor. Just go and buy eye drops from the pharmacy and treat yourself!”
Believe in traditional medicine	“Many people in our community believe modern medicines are less effective in treating eye problems than traditional medicines.”

The proposed solution to address both the barriers was to disseminate eye health knowledge through (1) organising eye health roadshows at the community level, (2) broadcasting eye health messages on social media via television or radios, and (3) sensitising the community through arts-based interventions such as songs, dramas, poems and themes. The goal is to sensitise the parents, teachers, children and community members with proper eye health knowledge. The stakeholders expected that children would be more likely to wear their eyeglasses if social stigma was reduced. Furthermore, children would perform better at school and have improved career opportunities with improved vision. The ultimate aim is to allow children to achieve their fullest potential with maximum health status.

### Consensus on the selection of art forms and eye health messages

Music and songs ranked at the top among the other proposed art forms. It is “the most effective method of disseminating informative knowledge to the community because it can quickly reach a large audience” (groups 1, 3, 4 and 5). The second choice was drama and the third was poetry because they are “useful as the content can remain for a long time within the audience” (groups 2 and 4). Cartoons ranked fourth because “children are more likely to be attracted to cartoons” (group 3) and therefore could be “a practical art form targeting the young population” (groups 1 and 2). The highest-ranked messages to be included in the ZANZI-ACE materials were that eye health is an individual responsibility and that seeking eye health services and maintaining good eye health lead to better well-being (table 5).

**Table 5:** Reported key messages to be included in the ZANZI-ACE music pieces

Rank	1	2	3	4	5	6
Themes	Self-responsibility	Seeking services	Well-being	Nutrition	Treatment	Superstition
**Key messages**	Everyone has the responsibility to take good care of their eyesight.	To seek health services when encountering eye problems.	Good eye health leads to a better quality of life.	Take nutritious food to gain healthy eyesight.	Some eye problems are treatable and preventable.	To avoid the element of superstition in handling eye conditions.
	Protect your eyes so that they may protect you.	Go to the hospital to seek treatment to improve your eyesight.	Eye health is essential for social well-being.	Eat nutritious food (fruits and vegetables) to enhance your eyes.	Protect your eyes by wearing eyeglasses (if you need them).	Avoiding superstition about eye disease.
	Reduce the use of mobile phones and computers to protect eye health.	Get proper advice from the doctor and treatment as soon as you know you have an eye problem.	Protect your child’s eye because it is a treasure in your life.		Eye problems are treatable.	
	Healthy eye begins with you.					

ZANZI-ACE, ZANZIbar Arts for Children’s Eyesight.

### Intervention, implementation strategy and the theory of change

The stakeholders perceived broadcasting songs/music containing eye health messages through a local radio station to be a well-accepted, reproducible and cost-efficient way to improve awareness regarding the importance of eye health among parents and children, a necessary step to increase eye health service uptake. The stakeholders also suggested that the intervention be tested for 3 months at the school and community levels before being implemented on a larger scale. They concluded that eye health education should be broadcast at the schools once during morning assembly, once during recess and once before school on Monday and Friday. At the community level, a popular local radio station would broadcast eye health education in the musical form three times a day to sensitise adults (table 6).

**Table 6:** Target audience, core messages, implementation strategy and duration of activities to be undertaken in the ZANZI-ACE pilot programme

Target audience	Core messages	Implementation strategy	Duration
Adults	Good eye health is important for children. Seek eye treatment at the hospital. Eat a balanced diet for good eye health. Protect your eyes from disease and harm.	Broadcast via a popular local radio station. Once every hour at peak time and once every 2 hours during off-peak.	3 months
Children		Broadcast at school once during morning assembly, once during recess and once before school on Monday and Friday.	3 months

ZANZI-ACE, ZANZIbar Arts for Children’s Eyesight.

At the end of the competition, five musical pieces were received. The top 3 musical pieces scored 673, 671 and 564 points, respectively. Subjective feedback from the judges reported that these pieces scored highest because (1) they contained clear, understandable, well-presented messages; (2) the lyrics are catchy; and (3) the music is well-arranged, good and attractive.

**Figure 1:**
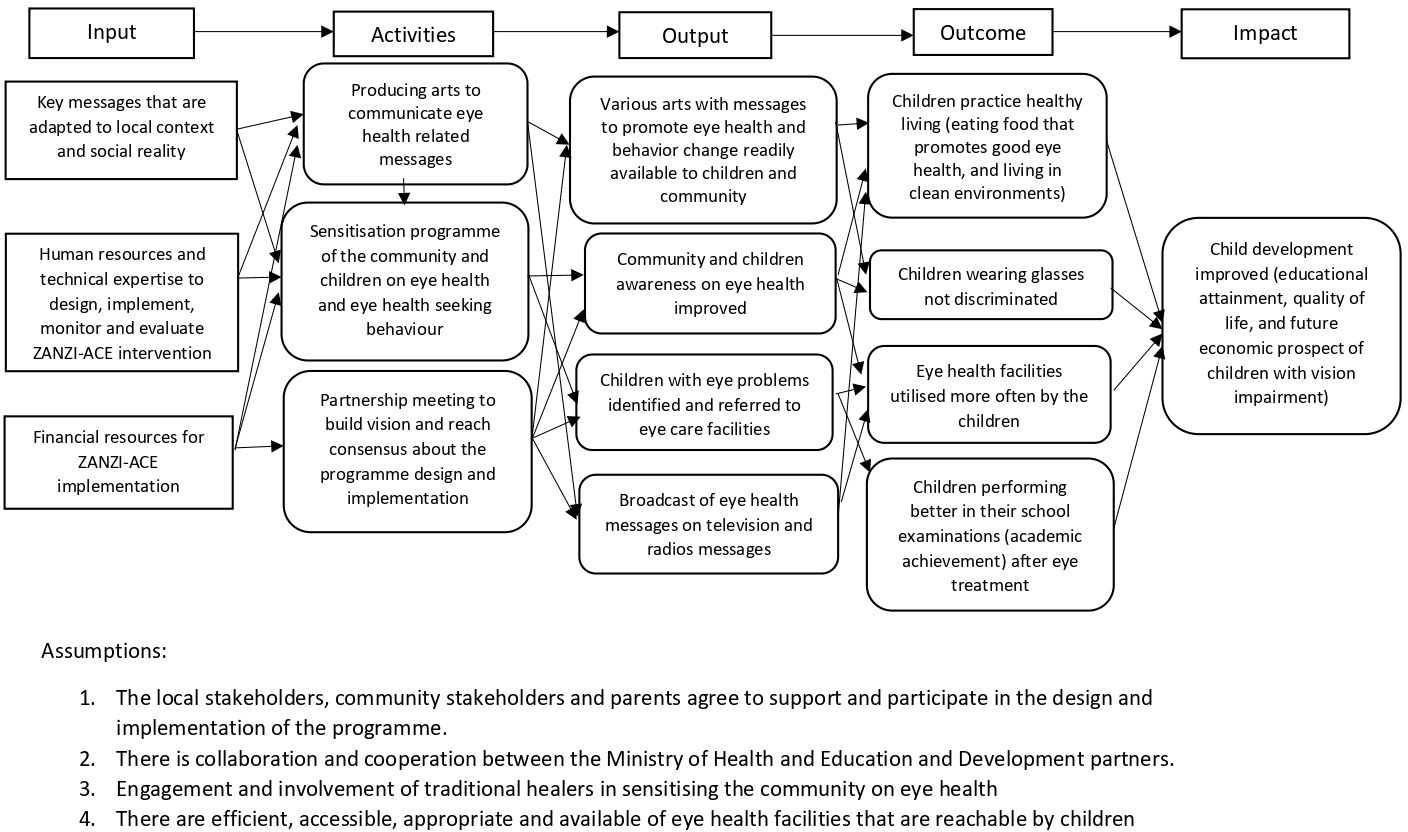
Visualisation of key elements of the co-created theory of change schema developed for ZANZIbar Arts for Children’s Eyesight (ZANZI-ACE).

The theory of change for the ZANZI-ACE programme was subsequently developed, with its inputs, outputs, activities, outcome and impact as depicted in figure 1. Based on the theory of change, the stakeholders also identified the key performance outcome indicators for the ZANZI-ACE programme. These include the proportion of children accessing eye health services, change in children’s and parents’ level of eye health knowledge and attitude, as well as treatment and spectacle compliance in children. Furthermore, implementation outcomes were identified, which broadly include intentionality (acceptability, feasibility, adoption), efficacy (impact of the content of the pieces to address sociocultural barriers) and responsiveness (how heritage and current musical forms contributed to the interventions’ efficacy and sustainability).

## Discussion

This article described the process and the outcomes of co-creating the materials, intervention and implementation strategy for ZANZI-ACE, which attempts to use music pieces in eye health education to improve child eye health service uptake in Zanzibar. Through a diverse group of local and international stakeholders, we identified the social and cultural barriers to child eye health service uptake, developed the key messages and music pieces for ZANZI-ACE, and formulated a theory of change and key indicator outcomes to measure the implementation performance of the ZANZI-ACE programme.

Co-creation processes inevitably face a range of challenges, not least of which include trust-building and achieving consensus among diverse perspectives and representatives. In the case of ZANZI-ACE, the co-creation process was further complicated by the severe restrictions on physical interaction and international travel posed by the COVID-19 pandemic. Ideally, the researchers would have preferred to conduct ethnographic research to observe the community’s dynamics and children’s environment.^18^ However, this was not possible as travel was extremely restricted. As an alternative, we used consultative workshop methods with diverse groups of community stakeholders to understand the local barriers to eye health.

This process affirmed some assumptions, such as the social stigma that inhibits children’s wear of eyeglasses and the lack of awareness of eye health. Our findings echoed those from Sudan,^32^ South Africa,^33^ India^34 35^ and Pakistan,^36^ where stigma towards eyeglasses discouraged uptake and wear. Grassroots knowledge was brought to the fore, such as the preference for traditional eye medicine in Zanzibar and that unaffordability is not the main barrier to uptake. Traditional medicine is deep-rooted in many developing countries, with over 3.5 billion people globally depending on traditional medicine (1997).^37^ In Zanzibar, traditional medicine has gained popularity since the 1980s^38 39^ and is regulated by the Zanzibar Traditional Medicine and Alternative Health Policy. As of 2015, over 99% of the population use traditional medicine because its use has always been embedded in the Zanzibari culture.^40^ We see that this is an opportunity to involve traditional healers in our intervention design as health programmes such as maternal care^41^ and mental health^42^ care have seen benefits from this approach.

We also adopted a ‘local’ approach to formulate key messages and co-create the music pieces. In this context, ‘local’ is defined as geographical location and social and cultural experiences. Using the competition format, we wanted to encourage like-minded talents to produce music and lyrics that would appeal to the community and invoke motivations and sensitisation locally. In addition, the selection of the final pieces for the ZANZI-ACE strategy by a diverse panel, which included religious leaders and traditional healers, may also increase the local appropriateness and acceptability of our materials.^43^

The interactions between the local and international stakeholders greatly highlighted the advantage of cross-cultural, multidisciplinary collaboration in the co-creation process.^44^ This interaction led to several unintended positive outcomes. First, the stakeholders highlighted that broadcasting the pieces at schools alone would not achieve the ZANZI-ACE goals. The intervention at the community level would be critical. Second, a high level of granularity is included in the implementation strategy. Third, a pilot will be important to test and modify our implementation strategy and the theory of change before large-scale implementation.

Balancing the needs of research and implementation for the ZANZI-ACE strategy was a complex process,^45^ which was occasionally in tension when developing the theory of change. While implementers emphasised the immediate effectiveness of the implementation in Zanzibar, researchers were also concerned with the design’s relation to subsequent studies to determine the strategy’s long-term effectiveness. Hence, the ZANZI-ACE theory of change consists of a list of outputs and outcomes measuring the strategy’s effectiveness and implementation. Although the list of indicators is long, it captured both stakeholders’ valid perspectives.

### Strengths and limitations

The strength of our co-creation process was that we strategically selected a diverse group of stakeholders to participate in every stage of the co-creation process. We used local talents to codevelop culturally appropriate music pieces. However, our approach also had its limitations. Providing reading materials to the stakeholders before the meeting may have also created bias in the stakeholders’ responses in the group discussions. It was at times challenging to ensure everyone’s opinions were heard and recorded in the large group dynamics of a workshop, where power dynamics are inevitably at play in achieving consensus. Intergenerational inclusion is a recognised challenge of such processes.^46^ As these limitations were anticipated, they were mitigated by recruiting well-trained and experienced local facilitators to conduct the meetings in the local language. While inclusive, workshops may not have enabled full comprehension and analysis of the range and complexities of sociocultural and contextual factors compared with robust ethnographic research methods.^47^ Furthermore, this paper only focused on the process, outcomes and lessons learnt from the co-creation processes and did not detail the critical interpretation of the music and musical content, which requires separate indepth analyses, followed by pretesting of the eye health messages, piloting the approach to identify the operational challenges and testing the ZANZI-ACE effectiveness in improving eye health service uptake and well-being of the children.

## Conclusion

The co-creation process and outcomes of the ZANZI-ACE programme demonstrate that engaging a diverse group of stakeholders in co-creation processes is critical to the development of locally relevant health programmes. We hope that the process and lessons learnt will contribute to knowledge in this area of research and that they may be useful to researchers who aspire to design innovative health programmes.^48^

## Data Availability

Data are available in a public, open access repository. Data are available at https://pure.qub.ac.uk/files/334460037/Workshop_notes_for_Zanzibar_Arts_for_Child_Eyesight_2_.xlsx.
