# Supplementary material for: Co-creating an arts-based eye health education strategy in Zanzibar: process, outcomes and lessons learnt
Source: BMJ Glob Health. 2022 Sep 6;7(9):e009317. doi: 10.1136/bmjgh-2022-009317 (PMC9454078; doi:10.1136/bmjgh-2022-009317)
Supplement: Supplementary data [file bmjgh-2022-009317supp001.pdf]

Study conceptualisation

1 How does this study address local research and policy priorities?

Eye health and health promotion are research topics identified by the local stakeholders in Zanzibar (shown in Chan et al. 2021 study), which aligns to the Zanzibar Health Policy.

2 How were local researchers involved in study design?

Yes. The local researchers are involved in the research and tool design with FO being the local Principal investigator for the study.

Research management

3 How has funding been used to support the local research team(s)?

61% of the budget was used to support the time, transport, organization of the local research team.

Data acquisition and analysis

4 How are research staff who conducted data collection acknowledged?

The research staff were all co-authors in the manuscript.

5 How have members of the research partnership been provided with access to study data?

One – the local principal investigator FO, as stipulated by the data management policy to ensure data safety.

6 How were data used to develop analytical skills within the partnership?

All local researchers were given tasks to analyse the meeting notes – framework development, extraction, coding, interpretation.

Data interpretation

7 How have research partners collaborated in interpreting study data?

All local researchers were given tasks to analyse the meeting notes – framework development, extraction, coding, interpretation.

Drafting and revising for intellectual content

8 How were research partners supported to develop writing skills?

Local researchers were paired with academics in the UK so that the local researchers practice the writing up of the manuscript. E.g. VFC was paired with FO, DB was paired with PG-P, CP-S and EM, DM is was paired with ACY, CG was paired with RG.

9 How will research products be shared to address local needs?

Reports were shared periodically with the local researchers through FO and EM. A ZANZI-ACE website is developed to share first hand information with local researchers.

Authorship

10 How is the leadership, contribution and ownership of this work by LMIC researchers recognised within the authorship?

All local researchers are included in the authorship where FO is the senior author.

11 How have early career researchers across the partnership been included within the authorship team?

All early career researchers (ACY, DM, CP-S, CG, PG-P) are co-authors on the paper.

12 How has gender balance been addressed within the authorship?

Six (DB, ACY, PG-P, DM, CG and FO) out of ten authors are female researchers.

Training

13 How has the project contributed to training of LMIC researchers?

PG-P was trained in ethics in conducting research in children, DM was trained in framework analysis, and FO and EM were trained in facilitation of workshop.

Infrastructure

14 How has the project contributed to improvements in local infrastructure?

Not applicable.

Governance

15 What safeguarding procedures were used to protect local study participants and researchers?

We were guided by the Save the Children: So you want to consult with children? A toolkit of good practice.
