# Supplementary material for: Co-creating an arts-based eye health education strategy in Zanzibar: process, outcomes and lessons learnt
Source: BMJ Glob Health. 2022 Sep 6;7(9):e009317. doi: 10.1136/bmjgh-2022-009317 (PMC9454078; doi:10.1136/bmjgh-2022-009317)

Supplemental file 2: Thematic diagram of the main key themes and subthemes emerged from workshop notes analysis

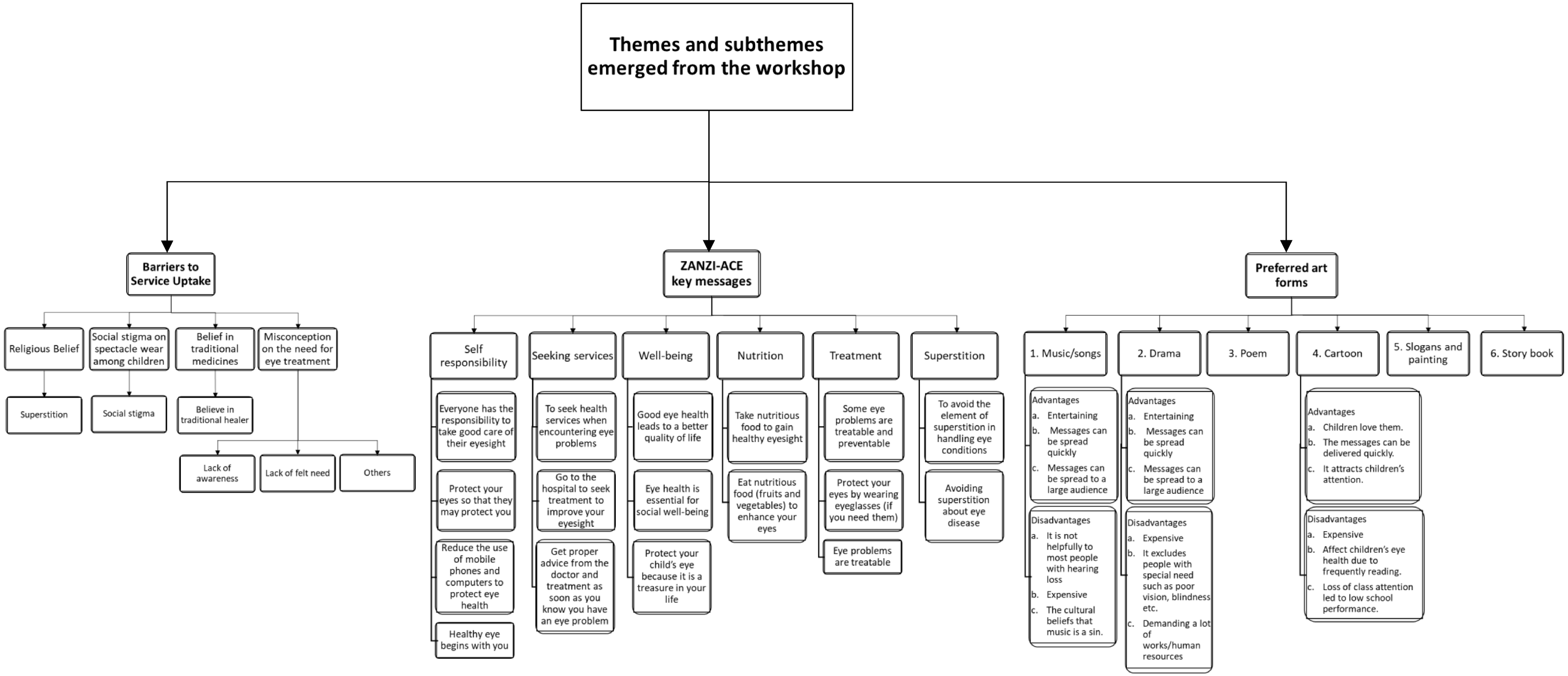

Supplement: Supplementary data [file bmjgh-2022-009317supp002.pdf]
